# The role of smoking habits in periodontal health and disease: A cross-sectional study

**DOI:** 10.18332/tid/225931

**Published:** 2026-09-29

**Authors:** Tuğba Şahin, Nazurah binti Nik-Eezammuddeen

**Affiliations:** 1 Division of Periodontology, Dentistry Faculty, Abant İzzet Baysal University, Bolu, Türkiye; 2 Division of Periodontology, Faculty of Dentistry, Universiti Teknologi MARA, Sungai Buloh, Malaysia

**Keywords:** gingivitis, periodontitis, periodontal diseases, smoking, tobacco products

## Abstract

**INTRODUCTION:**

Smoking is a major environmental factor in oral and periodontal tissue deterioration. The aim of this study was to investigate the association of cigarette smoking with periodontal health and disease severity.

**METHODS:**

The Periodontology Clinic at Bolu Abant İzzet Baysal University conducted a cross-sectional study from 2024 to 2026. Participants (aged ≥18 years) without systemic diseases and with no periodontal treatment within 6 months, were eligible. Antibiotic and anti-inflammatory drug users, pregnant or breastfeeding women, diabetics, waterpipe smokers, and chemotherapy and radiotherapy patients were excluded. Pack-years were computed by multiplying daily cigarette use by smoking years. Current periodontal status was determined using the 2017 World Workshop classification and 2023 EFP criteria. Statistical analyses were performed using SPSS 27.0 with t-tests, chi-squared tests, and logistic regression, with significance set at p<0.05.

**RESULTS:**

A total of 484 participants (242 current smokers and 242 non-smokers) attended the Periodontology Clinic of Bolu Abant İzzet Baysal University. While the overall distribution of periodontal diagnoses did not differ significantly (p=0.449), smokers exhibited more rapid progression of periodontitis (Grade C: 40.4% vs 11.5% in non-smokers, p<0.05). Smokers presented with significantly higher plaque index (PI) (p=0.024) and lower bleeding on probing values (p=0.028). Furthermore, longer smoking duration and higher pack-years were strongly associated with periodontitis severity (p=0.001). Multivariable logistic regression analysis revealed that female gender (AOR=2.51; 95% CI: 1.70–3.98, p=0.001) gingival recession (AOR=1.76; 95% CI: 1.05–2.94, p=0.001) were the strongest independent factors associated with smoking status, while each unit increase in PI was associated with 42% lower odds (AOR=0.58; 95% CI: 0.37–0.90, p=0.015).

**CONCLUSIONS:**

Smoking was associated with periodontal status and differences in disease severity and grade distribution. Additionally, tobacco exposure accelerated plaque and periodontal problems. These findings underscore the need to cease smoking for periodontal care.

## INTRODUCTION

Healthy gingiva is characterized by a periodontium with intact integrity, <10% clinical bleeding on probing (BOP), below 10% and a periodontal pocket depth (PPD) ≤3 mm^1^. Periodontal diseases affect the periodontium, which surrounds teeth, including the gingiva, alveolar bone, cementum, and periodontal ligament^2^. Gingivitis is characterized by inflammation that remains confined to the gingival tissues without affecting the periodontal tissues^1^. Periodontitis is a chronic multifactorial inflammatory disease associated with dysbiotic microbial dental plaque, which is characterized by progressive destruction of tooth-supporting tissues. It primarily involves clinical attachment loss, radiographically assessed alveolar bone loss on radiographic examination, periodontal pocket formation, gingival bleeding, and loss of periodontal tissues^3^.

Periodontal disease is an opportunistic infection that involves interactions resulting from the interactions between etiological factors (dental plaque, etc.) and host responses, which can be modulated by genetic, environmental, and acquired risk factors^4^. Another risk factor for periodontitis is smoking^5^, with tobacco use being a crucial currently recognized important risk factor for periodontal disease given that it adversely affects both the development of the disease and its treatment strategies. Exposure to tobacco products impairs oral microcirculation, which in turn aggravates periodontal disease^6^. Further, smoking has been shown to exacerbate the prevalence and progression of periodontitis^7^. Moreover, smoking cessation has been shown to improve periodontal health and reduce gingival inflammation^8^. Smoking adversely affects oxidative stress markers and inflammatory cytokines^9^. The harmful effects of smoking have been shown to be dose-dependent^10^. Periodontal classification was revised in 2017, and a stage-grade system was introduced. Smoking status serves as a grade modifier in determining the grade^3^. There has been extensive research on the effects of smoking on the periodontium^5,11^; however, the association of smoking with periodontal status remains relatively unclear^12^. Although smoking is a well-established risk factor for periodontitis, its association with periodontal state according to the 2017 World Workshop classification and smoking dose has not been completely examined.

The null hypothesis of this study was that there is no significant difference in the prevalence and severity of periodontal disease between smokers and non-smokers. Accordingly, this study aimed to examine the relationship between smoking and periodontal disease and health as classified according to the 2017 Classifications of Periodontal Disease.

## METHODS

### Study design

The cross-sectional study was conducted at the Periodontology Clinic of Bolu Abant İzzet Baysal University, Faculty of Dentistry, Bolu, Türkiye, from December 2024 to April 2026. The study recruited consecutive qualified clinic visitors. The manuscript follows the Strengthening the Reporting of Observational Studies in Epidemiology guidelines (STROBE)^13^.

### Exposure assessment

Current smokers were defined as active smokers who had smoked ≥100 cigarettes in their lifetime and continued smoking. Non-smokers consisted of never smokers or former smokers who had smoked <100 cigarettes in their lifetime.

### Inclusion and exclusion criteria

The inclusion criteria were: age ≥18 years, lacking systemic diseases that could affect periodontal or general health, and no history of periodontal treatment within the past 6 months. The exclusion criteria were: individuals using antibiotics or anti-inflammatory drugs; pregnant or lactating women; patients with diabetes; individuals who smoked waterpipe or chewed tobacco (smoker group); and individuals undergoing chemotherapy or radiotherapy.

### Sample size and recruitment

Based on the results of the power analysis performed using the G*Power program (G*Power 3.1 software; Heinrich Heine University, Düsseldorf, Germany), a t-test (difference between two independent means) was conducted on data from the two groups by smoking status. With an alpha error probability (α) of 0.05, an effect size (d) of 0.30, and a power (1-β) of 0.95, it was determined that a minimum of 484 participants (n=242 per group) would be sufficient. The study sample size was calculated as described by Costa et al.^14^.

Five hundred fifty-three patients were referred to the Periodontology Clinic at Bolu Abant İzzet Baysal University; of these, 484 were included in the study based on the inclusion and exclusion criteria. Based on the smoking history, participants were classified as current smokers (test group, n=242) and non-smokers (control group, n=242).

### Variables

The key variables of this study were smoking status and periodontal status. Smoking status was defined as non-smoker and smoker based on self-reported smoking history. Information regarding the daily number of cigarettes smoked and the duration of smoking in years, with the pack-years being calculated, was collected accordingly. Additionally, the plaque index (PI), the gingival index (GI), the periodontal probing depth (PPD), bleeding on probing (BOP), gingival recession (GR), and clinical attachment level (CAL) of all participants were evaluated and recorded. Other variables included demographic characteristics (age, sex, height, and weight), medication use (yes, no), smoking-related characteristics (daily cigarette consumption, smoking duration [years], and cumulative smoking exposure [pack-years]), and clinical periodontal parameters, including PI, GI, PPD (mm), GR (mm), CAL (mm), and BOP (% of sites). A calibrated UNC-15 periodontal probe (PCP-15; Hu-Friedy, Chicago, IL, USA) was used to take these readings. All clinical data were measured and documented by a single calibrated clinician following a standardized periodontal examination technique. Periodontal health status (periodontal health, gingivitis, and periodontitis) was assessed by one clinician (TŞ) based on the 2017 World Workshop on Classifying Periodontal and Peri-Implant Diseases and Conditions and 2023 European Federation of Periodontology Guidelines. Additionally, patients with periodontitis were classified by staging and grading the disease^3^.

### Statistical analysis

Statistical analyses were performed using IBM SPSS Statistics for Windows, Version 27.0 (IBM Corp., Armonk, NY, USA; IBM Corp., 2020)^15^. Descriptive statistics were summarized as frequencies (n) and percentages (%) for categorical variables, and as mean and standard deviation (SD) for continuous variables. The normality of data distribution was assessed using the Kolmogorov–Smirnov and Shapiro–Wilk tests. Although the data exhibited a non-normal distribution, parametric tests were preferred due to the large sample size (n=484), satisfying the Central Limit Theorem.

Bivariate comparisons of periodontal parameters (PI, GI, CAL, BOP, PPD, and GR) and demographic factors between the groups were performed using the independent samples t-test. For evaluations involving multiple subgroups based on periodontal health status, one-way analysis of variance (ANOVA) was utilized, followed by the Bonferroni post hoc test for pairwise comparisons. Categorical variables were analyzed using the chi-squared test.

To evaluate the independent relationship between smoking status, demographics, and periodontal conditions, a multivariable binary logistic regression analysis was conducted. Covariates were selected for inclusion in the regression model based on their clinical relevance, prior literature support, and significance in bivariate screening. Model outputs were reported as adjusted odds ratios (AORs) with their corresponding 95% confidence intervals (CIs) to provide precise effect estimates rather than relying solely on significance levels.

## RESULTS

Based on smoking history, participants were classified as current smokers (n=242) or non-smokers (n=242).

### Demographic characteristic

Among the participants included, 57% were female, and 43% were male. Regarding education level, 19.4%, 7.6%, 34.1%, and 38.8% had completed primary, secondary, high school, and university education, respectively. Further, 49.8% of the participants were employed. Regarding health conditions, 21.3% of the participants had a chronic disease. Additionally, 24.2% regularly used medication (Table 1).

**Table 1:** Distribution of periodontal health status, periodontal disease stage, and grade by smoking status among adults attending the Periodontology Clinic of Bolu Abant İzzet Baysal University, Türkiye, 2024–2026 (N=484)

*Characteristics*		*Smoker* *(N=242)* *n (%)*	*Non-smoker* *(N=242)* *n (%)*
**Sex**	Female	112 (46.3)	164 (67.8)
	Male	130 (53.7)	78 (32.2)
	χ^2^=22.797, p=0.001*		
**Age** (years)	Mean ± SD	34.38 ± 12.02	37.14±14.61
	t= -2.267, p=0.024*		
**BMI** (kg/m^2^)	Mean ± SD	25.66 ± 5.40	26.41 ± 5.73
	t= -1.490, p=0.137		
**Education level**	Primary school	39 (16.1)	55 (22.7)
	Secondary school	25 (10.3)	12 (5.0)
	High school	96 (39.7)	69 (28.5)
	Bachelor’s	82 (33.9)	106 (43.8)
	χ^2^=14.773, p=0.002*		
**Working**	Yes	154 (63.6)	87 (36.0)
	No	88 (36.4)	155 (64.0)
	χ^2^=37.100, p=0.001*		
**Illness**	Yes	46 (19.0)	57 (23.6)
	No	196 (81.0)	185 (76.4)
	χ^2^=2.351, p=0.090		
**Medication use**	Yes	53 (21.9)	64 (26.4)
	No	189 (78.1)	178 (73.6)
	χ^2^=1.364, p=0.243		
**Periodontal status**	Gingivitis	138 (57.0)	150 (62.0)
	Periodontitis	52 (21.5)	42 (17.4)
	Periodontal health	52 (21.5)	50 (20.7)
	χ^2^=1.609, p=0.449		
**Grade**	A	6 (11.5)	24 (57.1)
	B	25 (48.1)	13 (31.0)
	C	21 (40.4)	5 (11.9)
	χ^2^=23.639, p=0.001*		
**Stage**	1	8 (15.4)	2 (4.8)
	2	15 (28.8)	18 (42.9)
	3	19 (36.5)	16 (38.1)
	4	10 (19.2)	6 (14.3)
	χ^2^=4.113, p=0.250		
**Stage-Grade**	1 A	4 (7.1)	2 (4.8)
	2 A	1 (1.8)	14 (33.3)
	3 A	1 (1.8)	6 (14.3)
	4 A	0 (0.0)	2 (4.8)
	1 B	3 (5.4)	0 (0.0)
	2 B	9 (16.1)	3 (7.1)
	3 B	10 (17.9)	6 (14.3)
	4 B	3 (5.4)	4 (9.5)
	1 C	1 (1.8)	0 (0.0)
	2 C	5 (8.9)	1 (2.4)
	3 C	8 (14.3)	4 (9.5)
	4 C	7 (12.5)	0 (0.0)
	Fishers exact test χ^2^=36.153, p=0.005*		

* p<0.05.

The mean age, height, body weight, and body mass index were 35.76 ± 13.43 years (range: 18–65), 1.68 ± 0.09 m, and 74.17 ± 15.53 kg, and 26.17 ± 5.33 kg/m^2^, respectively. The non-smoker group had a significantly higher mean age than the smoker group (p<0.05). Mean daily cigarette consumption, smoking duration, and annual cigarette pack consumption were 12.9 ± 8.8 cigarettes, 12.4 ± 10.1 years, and 176.1 ± 198.2 pack-years, respectively.

### Between-group comparison of periodontal status

Comparing the periodontal health profiles of smokers (n=242) and non-smokers (n=242), no statistically significant difference was observed between the groups in overall diagnosis distributions (gingivitis, periodontitis, and healthy condition) (χ^2^=1.609, p=0.449). In both groups, gingivitis constitutes the most frequently observed clinical condition. However, in the periodontitis group, when classified according to the progression rate (Grade) and severity (Stage) of the disease, differences between smokers and non-smokers are observed (p<0.05). The majority of non-smoking individuals (57.1%) fall into the slow-progressing (Grade A) category, while among smokers, this rate decreases to 11.5%, and the prevalence of Grade C, defined as the ‘fast-progressing’ form, is higher (40.4%).

When examined by disease stage, although Stage 3 was prevalent in both groups, the frequency of the most severe stage, Stage 4, was numerically higher in smokers (19.2%) than in non-smokers (14.3%). However, this does not result in a statistically significant difference between the groups (χ^2^=4.113, p=0.250). Similarly, in the Stage-Grade interaction, the combination of Stage 4 Grade C was not observed in the non-smoking group (0.0%), while the diagnosis occurred in 12.5% of the smoking individuals (Table 1). In contrast, the Stage 2 Grade A combination, which is a low-severity and slow-progressing condition, is observed in 33.3% of non-smokers, while this rate drops to 1.8% among smokers (Supplementary file).

### Comparison of smoking status according to indices

The smoker group had significantly higher plaque index values than the non-smoker group (p=0.024). Additionally, the non-smoker group had significantly higher bleeding on probing values than the smoker group (p=0.028) (Table 2).

**Table 2:** Comparison of periodontal indices by smoking status among adults attending the Periodontology Clinic of Bolu Abant İzzet Baysal University, Türkiye, 2024–2026 (N=484)

		*n*	*Mean*	*SD*	*t*	*p*
**Plaque index**	Smoker	242	0.90	0.65	2.267	0.024*
	Non-smoker	242	0.77	0.58		
**Gingival index**	Smoker	242	0.66	0.61	0.568	0.570
	Non-smoker	242	0.63	0.58		
**Periodontal pocket depth**	Smoker	242	2.65	0.65	0.336	0.737
	Non-smoker	242	2.63	0.65		
**Bleeding on probing**	Smoker	242	25.27	18.99	-2.203	0.028*
	Non-smoker	242	29.45	22.60		
**Gingival recession**	Smoker	242	0.12	0.43	-0.523	0.601
	Non-smoker	242	0.14	0.40		
**Clinical attachment level**	Smoker	242	2.78	0.89	0.052	0.958
	Non-smoker	242	2.77	0.88		

* p<0.05.

### Comparison of cigarette consumption according to periodontal conditions

Daily cigarette consumption differed significantly across periodontal status groups (p=0.049), with higher values among individuals with periodontitis (8.18 ± 10.97 cigarettes) and lower values among those with periodontal health (5.05 ± 7.64 cigarettes). Moreover, smoking duration also differed significantly between groups (p=0.001), with longer durations observed in individuals with periodontitis (9.44 ± 11.00 years) and shorter durations in those with healthy periodontal tissues (4.02 ± 6.61 years).

Similarly, annual pack-years of smoking showed a statistically significant difference across periodontal status groups (p=0.001), with higher values observed in individuals with periodontitis (145.46 ± 218.21 pack-years) and lower values in those with periodontal health (41.60 ± 89.29 pack-years) (Table 3).

**Table 3:** Comparison of daily cigarette consumption, smoking duration, and pack-years according to periodontal status of adults attending the Periodontology Clinic of Bolu Abant İzzet Baysal University, Türkiye, 2024–2026 (N=484)

		*n*	*Mean*	*SD*	*F*	*p*
**Daily cigarettes**	Gingivitis	288	6.38^a^	8.61	3.031	0.049*
	Periodontitis	94	8.18^b^	10.97		
	Periodontal health	102	5.05^c^	7.64		
	Total	484	6.45	8.97		
**Duration** (years)	Gingivitis	288	5.93^a^	9.49	8.642	0.001*
	Periodontitis	94	9.44^b^	11.00		
	Periodontal health	102	4.02^c^	6.61		
	Total	484	6.21	9.43		
**Pack-years**	Gingivitis	288	85.80^a^	161.14	10.073	0.001*
	Periodontitis	94	145.46^b^	218.21		
	Periodontal health	102	41.60^c^	89.29		
	Total	484	88.07	165.42		

F: one-way ANOVA analysis. Superscripted letters a, b, and c indicate group comparisons; groups sharing the same letter do not differ significantly.

* p<0.05.

### Comparison of cigarette consumption with periodontitis classification

Smoking duration did not differ significantly across periodontitis stages (F=1.857, p=0.057); however, smoking duration was longer in participants with Stage C periodontitis and shorter in those with Stage A periodontitis. Moreover, the Stage A group had significantly different smoking durations from the other groups.

Daily cigarette consumption, smoking duration, or pack-years did not significantly differ according to the periodontitis stage and grade (F=1.857, p=0.057; F=1.832, p=0.061; F=1.558, p=0.127).

### The relationship between smoking status, periodontal condition, and demographic characteristics

According to the results of the binary logistic regression analysis, smokers are the reference category, and periodontal and demographic variables associated with smoking status are presented. As a result of the binary logistic regression analysis, it was determined that the model is statistically significant (χ^2^=51.115; df =9; p<0.001). The explanatory power of the model was 13.4%, based on the Nagelkerke R² value. Likelihood ratio tests indicated that age, gender, PI, BOP, and GR were significant factors associated with smoking status (Table 4).

**Table 4:** Binary logistic regression analysis of factors associated with non-smoking status of adults attending the Periodontology Clinic of Bolu Abant İzzet Baysal University, Türkiye, 2024–2026 (N=484)

*Variables*	*AOR*	*95% CI for exp(B)*		*p*
		*Lower*	*Upper*	
Intercept				0.544
Age	1.02	1.00	1.04	0.018*
Plaque index	0.58	0.37	0.90	0.015*
Gingival index	1.28	0.79	2.04	0.307
Periodontal pocket depth	0.99	0.73	1.25	0.998
Bleeding on probing	1.01	1.00	1.02	0.011*
Gingival recession	1.76	1.05	2.93	0.001*
Clinical attachment level	0.99	0.81	1.20	0.998
Sex: Female	2.51	1.70	3.98	0.001*
Sex: Male				
Medication use: Yes	0.54	0.51	1.37	0.198
Medication use: No				

AOR: adjusted odds ratio; calculated via multivariable binary logistic regression (Enter method). The model was simultaneously adjusted for age, gender, medication use, PI, GI, BOP, GR, CAL and PPD. Cox and Snell R^2^=0.100, Nagelkerke R^2^=0.134, χ^2^=51.115, df=9, p=0.001. Reference category: smoker.

* p<0.05.

According to the analysis results, each unit increase in age was associated with a 2% increase in the adjusted odds of the related condition (AOR=1.02; 95% CI: 1.00–1.04, p=0.018,). Among the periodontal indicators, the PI was significantly associated with lower odds of the outcome (AOR=0.58; 95% CI: 0.37–0.90, p=0.015). Accordingly, each unit increase in the PI was associated with approximately 42% lower odds of the condition (1-0.58=0.42). On the other hand, each unit increase in the BOP was associated with a 1.4% increase in the odds (AOR=1.01; 95% CI: 1.00–1.02, p=0.011). Among the clinical parameters, GR stands out as one of the strongest factors associated with risk; a one-unit increase in GR increases the probability by 1.76 times. Among the clinical parameters, GR was identified as a significant factor; a one-unit increase in GR was associated with 1.75 times higher odds of the condition (AOR=1.76; 95% CI: 1.054–2.935, p=0.001). Regarding the demographic variable of gender, being female was associated with 2.51 times higher odds of the condition compared to the reference group (male), and this association was statistically significant (AOR=2.51; 95% CI: 1.70–3.98, p<0.001). The variables included in the model, such as height, weight, GI, PPD, CAL, and medication use, were not significantly associated with the outcome (p>0.05).

## DISCUSSION

This study evaluated the association between smoking habits and periodontal disease and overall periodontal health. The results showed that a higher prevalence of periodontal health was observed in non-smokers, whereas smokers exhibited a higher prevalence of periodontitis. Additionally, differences were observed between groups in terms of plaque accumulation and BOP.

Compared with non-smokers, current smokers have a higher risk of periodontal disease^16^. Although smoking is not considered an absolute contraindication for periodontal treatment, it increases the risk of less favorable therapeutic outcomes^17^. Former smokers who had quit for ≤5 years showed a higher and lower risk than non-smokers and current smokers, respectively. Smoking cessation allows partial recovery of periodontal health^16^. Smoking was significantly associated with the severity of periodontal disease. This relationship was most apparent in former smokers, who demonstrated the highest proportion of advanced periodontal stages. A significant relationship was observed between smoking status and the extent of periodontal destruction. Among smokers (32.8%), non-smokers (37.5%), and passive smokers (28.4%), most participants exhibited gingival inflammation consistent with clinical gingival health in an intact periodontium^8^. Notably, users of heated tobacco products, cigarettes, or both, as well as former users, have shown a significantly higher prevalence of periodontal disease than never users^18^. In addition, the higher proportion of non-smokers recognizing the association between smoking and periodontal disease compared with smokers^19^. Smoking status was strongly associated with periodontal condition, with current smokers exhibiting the highest prevalence of periodontitis, followed by former smokers and never smokers. The proportion of individuals with periodontitis increased progressively from never smokers to former and current smokers, highlighting a clear dose–response relationship between tobacco exposure and periodontal disease occurrence^20^. Consistent with previous reports, smokers were more prevalent among individuals with periodontitis, whereas non-smokers were more common among those with periodontal health.

Regarding the periodontium, smoking exacerbates the prevalence and severity of periodontal disease; further, it attenuates the effectiveness of periodontal therapy^21^. Long-term smoking is strongly associated with poor periodontal health, which reflects its cumulative detrimental effects on the periodontal tissue^16^. Cigarette smoking is a well-established risk factor for periodontal disease. It accelerates periodontal destruction by damaging periodontal ligament cells, promoting alveolar bone loss, and impairing bone remodeling. Furthermore, cigarette smoke may enhance bacterial biofilm formation and modulate microbial metabolism, which contributes to disease progression^22^. Šutej et al.^23^ reported that smokers have significantly greater PPD than non-smokers, with the PPD being positively correlated with the extent of cigarette smoking. Smokers showed more supragingival calculus, whereas non-smokers exhibited greater BOP, which indicated between-group differences in the inflammatory profiles^23^. Compared with non-smokers, smokers showed higher mean PPD, clinical attachment loss, and PI values, which indicates exacerbated periodontal destruction^24^. Vohra et al.^25^ observed that the PI and PPD were greater in conventional cigarette smokers than in electronic cigarette users and non-smokers. Another study declared that cigarette smoking is associated with increased severity of periodontal disease, as evidenced by a greater GI, lower PI, and greater clinical attachment loss^26^. This isolated population showed a high prevalence of increased PPD. Additionally, behavioral risk factors have been shown to significantly contribute to increased PPD values^27^. Marruganti et al.^28^ observed a higher proportion of individuals with residual PPD ≥5 mm among smokers than among non-smokers^28^. Compared with non-smokers, smokers showed significantly deeper and greater clinical attachment loss, which further highlights the detrimental effect of tobacco use on periodontal tissue integrity^29^. Smokers showed greater periodontal destruction than non-smokers, with a PPD of 52% higher and a mean CAL of 70% higher. In contrast, BOP has been reported to occur in 45% and 78% of sites in smokers and non-smokers, respectively^30^. Preus et al.^31^ reported that current smokers showed more severe periodontal destruction at baseline than former and never smokers. Although the between-groups difference in PPD and clinical attachment loss persisted after 12 months, it did not retain statistical significance, which suggests a reduced long-term impact of smoking following therapy^31^. Rosa et al.^32^ declared that smoking cessation reduced the PPD and clinical attachment loss following non-surgical periodontal therapy. Our findings are consistent with previous studies^24,30^; in particular, GR and BOP showed positive associations, whereas PI was inversely associated with smoking status.

Smoking has dose-dependent harmful effects, which are especially severe in younger individuals. Additionally, smoking has been associated with periodontitis recurrence during periodontal maintenance, with the risk increasing in dose-dependent manner. Heavy smokers (≥10 cigarettes per day) experience greater disease progression^33^; further, the risk increases with the number of pack-years, indicating a dose-dependent relationship^16^. Smoking pack-years have been significantly correlated with PPD and clinical attachment loss in both e- cigarette and cigarette smokers. Compared with e-cigarette smokers, cigarette smokers showed greater periodontal worsening. Additionally, smoking is a prognostic factor for clinical attachment loss in both cigarette and e-cigarette smokers^34^. Long-term cigarette use can have adverse effects on the mucous membranes^35^. Smoking duration is positively correlated with the severity of periodontal damage as well as PPD and clinical attachment loss values^30^. Compared with non-smokers, smokers presented significantly lower plaque and papillary bleeding indices than non-smokers. This reduction in bleeding tendency among smokers, despite comparable plaque accumulation, may be attributed to the vasoconstrictive effects of nicotine, which can mask the underlying gingival inflammation^29,6^. Duarte et al.^36^ demonstrated that tobacco cessation significantly reduces the risk of periodontitis and tooth loss. Further, the detrimental effects of smoking on periodontal tissues progressively diminish over the years since smoking cessation^36^. In this study, daily cigarette consumption, smoking duration, and pack-years were greater in participants with periodontitis than in those with periodontal health. However, these smoking-related parameters did not significantly differ across periodontitis stage and grade.

### Limitations

This study has several limitations. First, there may be selection bias given that the sample is not representative of the general population. Second, there may be recall bias given that self-reporting of smoking habits, including oral hygiene and socioeconomic status. Third, variations in clinical measurements may introduce information bias. Fourth, the study design limits the ability to determine causality. Nonetheless, the findings provide valuable insights into the relationship between smoking habits and periodontal health. Further, the inclusion of both smokers and non-smokers, as well as the use of standardized clinical periodontal parameters, strengthened the reliability of the comparisons and highlighted smoking as a potential modifiable risk factor for periodontal disease.

## CONCLUSIONS

The findings of this study indicate that smoking was associated with periodontal status and differences in disease severity and grade distribution. Additionally, smoking-related parameters such as daily cigarette consumption, duration, and pack-years were higher in individuals with periodontitis, although these did not differ significantly across disease stage and grade. Logistic regression analysis identified age, gender, GR, and BOP as significant factors associated with smoking status. However, due to the cross-sectional design, no causal inference can be made. Smoking cessation may be considered an important component in the management of periodontal diseases^36^, and further longitudinal studies are needed to clarify these associations.

## Supplementary Material



## Data Availability

The data supporting this research are available from the authors on reasonable request.
